# Dermocosmetic Potential of *Punica granatum*: A Systematic Review of Bioactive Compounds and Skincare Applications

**DOI:** 10.3390/antiox15030332

**Published:** 2026-03-06

**Authors:** Nerea Pons-Rocamora, Enrique Barrajón-Catalán, María Herranz-López, Vicente Micol, Francisco Javier Álvarez-Martínez

**Affiliations:** 1 Instituto de Investigación, Desarrollo e Innovación en Biotecnología Sanitaria de Elche (IDiBE), Universidad Miguel Hernández (UMH), 03202 Elche, Spainmherranz@umh.es (M.H.-L.); f.alvarez@umh.es (F.J.Á.-M.); 2 CIBER, Fisiopatología de la Obesidad y la Nutrición, CIBERobn, Instituto de Salud Carlos III, 28029 Madrid, Spain

**Keywords:** aging, antioxidant, dermocosmetic, extract, polyphenol, pomegranate, skin

## Abstract

Background: *Punica granatum* L. (pomegranate) is a medicinal plant traditionally used for its antimicrobial and antioxidant effects. Recent evidence supports its expanding applications in dermatology and dermocosmetics. Purpose: This systematic review aimed to evaluate the skin-related biological activities of pomegranate extracts, identify the key bioactive compounds involved, and elucidate the underlying molecular mechanisms relevant to skin health and aging. Methods: A total of 732 studies were screened using AIReviewer clustering. Fifty-four original articles were selected on the basis of inclusion criteria prioritizing molecular evidence, *in vitro* and *in vivo* assays, and clinical relevance. Results: Pomegranate extracts exhibit a broad range of dermocosmetic properties, including antioxidant, anti-inflammatory, antibacterial, wound healing, moisturizing, photoprotective, and collagen-preserving effects. These effects are primarily attributed to ellagitannins (punicalagin and punicalin), ellagic and gallic acid, triterpenoids (oleanolic, maslinic, and asiatic acids), flavonoids (quercetin and catechins), anthocyanins, and fatty acids (punicic acid). Pomegranate extracts modulate oxidative stress by scavenging reactive oxygen species and upregulating Nrf2-mediated antioxidant pathways. They inhibit matrix metalloproteinases (MMP-1 and MMP-3), suppress proinflammatory cytokines (TNF-α and IL-6), and stimulate fibroblast proliferation, extracellular matrix remodeling, and hyaluronic acid synthesis. Their photoprotective activity provides enhanced UVB resistance and higher SPF values. Recent advances in fermentation and nanotechnology have been shown to increase the bioavailability and stability of compounds found in pomegranate, offering new formulation strategies. Conclusions: Pomegranates are a promising source of multifunctional phytochemicals with validated dermocosmetic applications. Their incorporation into advanced delivery systems may increase their therapeutic potential for skin protection, regeneration, and antiaging interventions.

## 1. Introduction

In recent years, there has been a notable upsurge in the inclination toward the utilization of natural products in the field of skin care. This trend shows steady growth as consumers increasingly perceive such products as safe and reliable. Consequently, the incorporation of natural products in topical preparations is among the most promising strategies in this domain [1].

**Figure 1:**
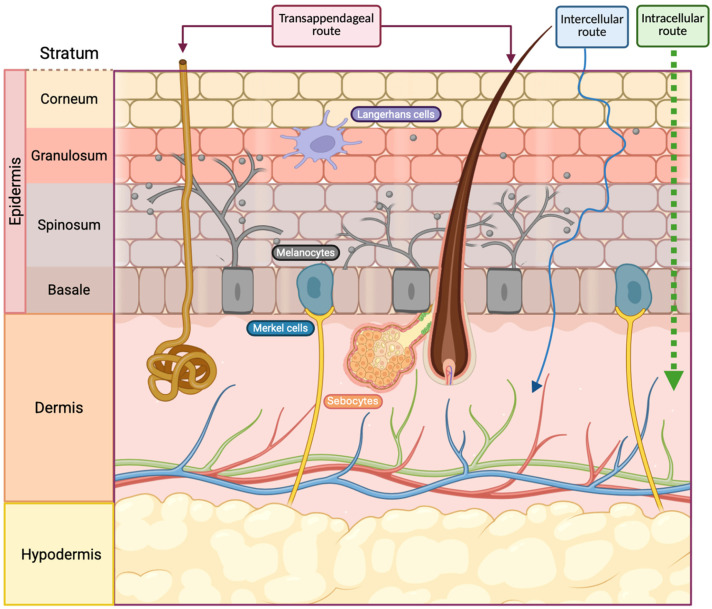
Different layers and sublayers of human skin and possible routes for permeation of compounds.

The skin has a total area of approximately 2 m^2^ and is acknowledged as the largest organ of the human body. It exhibits a lipophilic characteristic and is equipped with a multitude of pores. The skin consists of three layers, namely, the epidermis, dermis and hypodermis, which serve as protective barriers against topically applied substances. The epidermis consists of stratified squamous keratinized epithelial cells, encompassing both keratinocytes and nonkeratinocytes [2], stratified into four sublayers, the stratum basale, stratum spinosum, stratum granulosum, and stratum corneum (Figure 1) [3]. The skin not only provides protection but also effectively guards against ultraviolet (UV) light and performs various immunological functions through the presence of melanocytes, Merkel cells, and Langerhans cells [4]. Furthermore, skin health is significantly influenced by the skin microbiota, a complex ecosystem of microorganisms that maintains the epithelial barrier and modulates local immune responses. Maintaining the homeostatic balance of this microbiome is essential, as its disruption can lead to various skin conditions and infections, highlighting the importance of antibacterial and antifungal properties in dermocosmetic ingredients [5].

Given its prominent visibility, the skin offers a constant reminder of the aging process [6] as well as playing crucial roles in the body. It produces vitamin D, activates hormones, and affects stress responses. Collagen, elastin, and hyaluronic acid are essential components of the skin that contribute to its strength, elasticity, and hydration. However, oxidative stress caused by an increase in reactive oxygen species (ROS) can cause premature aging, leading to wrinkles, inflammation, and pigmentation disorders [7].

In this context, there are two types of skin aging: natural aging over time and premature aging caused by environmental factors such as sun exposure. Sun exposure, specifically UV radiation, stimulates the formation of free radicals, which induce oxidative stress, leading to the damage of collagen and elastin fibers through cross-linking, fragmentation, and amino acid modification, ultimately impairing their structural integrity and function. This damage to the skin’s collagen and elastin results in visible signs of aging [6]. The inhibition of elastase, collagenase, and tyrosinase enzymes is a key strategy to prevent both premature aging and hyperpigmentation. Moreover, the skin’s ability for wound healing and tissue repair is a fundamental aspect of its protective function, often requiring external support from bioactive compounds to accelerate regeneration and prevent chronic injuries [8]. Furthermore, owing to its highly cross-linked structure and minimal turnover, the half-life of elastin in the adult human dermis is estimated to be approximately 70 years [9]. Consequently, prevention of damage to elastin is essential for mitigating skin laxity.

Pollution from airborne particles can also contribute to skin aging through the generation of ROS [10,11]. Oxidative damage is not limited to protein alterations; it also contributes to the generation of a proinflammatory environment and DNA damage. Natural antioxidants have shown promise in protecting the skin from the damaging effects of oxidative stress [12]. These findings form the basis for the development of cosmetic products with bioactive antioxidant ingredients designed to overcome these negative effects and provide both beauty and health benefits.

Plant extracts have been demonstrated to contain a large number of bioactive molecules of dermocosmetic interest. The utilization of plant extracts and herbs for their medicinal properties can be traced back to ancient civilizations, with historical records from both China and Egypt still in existence [6]. In the search for potent botanical solutions to counteract oxidative aging, certain species have emerged as superior sources of protective metabolites. Notably, among the cultivated fruits, the pomegranate (*Punica granatum* L.) stands out as an exceptional resource. This study evaluates the biological potential of extracts derived from its juice, seeds, flowers, and peel, all of which possess numerous molecules with notable health-promoting activities [13]. The bioactivity of pomegranate extracts is attributed to a rich profile of compounds, including polyphenols such as punicalagins and ellagic acid. These constituents affect the skin through specific mechanisms, including the modulation of key enzymes such as matrix metalloproteinases (MMPs) and hyaluronidase, which are involved in extracellular matrix degradation. Furthermore, pomegranate bioactives can influence cellular pathways involved in antioxidant defense and inflammatory responses by dermal cells. Historically utilized in traditional medicine and dermocosmetics [14,15], pomegranate extracts are the subject of this review. The bioactivity of pomegranate extracts is attributed to a rich profile of compounds, including polyphenols such as punicalagins and ellagic acid [16]. These constituents not only act as antioxidants but also exhibit significant potential in accelerating wound healing and acting as potent enzyme inhibitors. Furthermore, pomegranate has demonstrated antimicrobial activity that may support skin health by modulating the microbiota and protecting against pathogens [5,17]. This study aims to critically analyze the literature on the dermocosmetic properties of pomegranate extracts to assess their true cosmetic potential and future prospects.

This review evaluates recent research on pomegranates, focusing on the dermocosmetic applications of their bioactive compounds and current technological innovations, aiming to provide a comprehensive synthesis of the available data.

## 2. Materials and Methods

### 2.1. Study Design and PRISMA Compliance

This systematic review was conducted in accordance with the Preferred Reporting Items for Systematic Reviews and Meta-Analyses (PRISMA) statement [18]. The methodology was designed to ensure a transparent and complete reporting of the evidence synthesis regarding the dermocosmetic properties of pomegranate extracts. The selection process, eligibility criteria, and data extraction followed the standardized phases of identification, screening, eligibility, and inclusion.

### 2.2. Literature Search and Analysis Powered by AIReviewer

This review was conducted by utilizing the capabilities of AIReviewer [19]. AIReviewer is composed of a series of Python (3.14.3) scripts coded into a Jupyter notebook that enables the classification of numerous scientific articles by clustering them on the basis of their content using natural language analysis. Once analyzed, information about the different articles is obtained in the form of global word frequency graphs, specific frequencies per cluster, word clouds, principal component analysis, Excel tables and time trend graphs (see AIReviewer Documentation (https://drive.google.com/file/d/1pGn-yzJ4K-NqBnOeIUqQffwuOc4ImHZZ/view?usp=sharing, accessed on 2 April 2024)).

**Figure 2:**
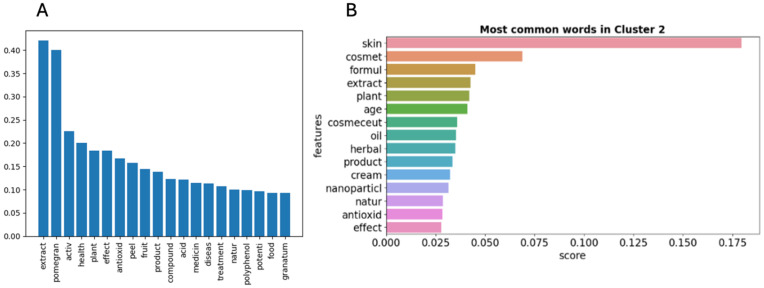
(**A**) Frequency bar graph with the 20 most frequent words and the total number of articles analyzed. (**B**) Frequency bar graph with the 15 most frequent words in article Cluster 2.

The initial selection of articles for review was conducted through a search process employing specific keywords, namely, “Pomegranate AND extract AND health OR cosmetic”, in Scopus. This search yielded 732 articles. AIReviewer was used to screen and classify the scientific articles related to pomegranate extracts and their dermocosmetic properties into clusters. The graphs presented in Figure 2 display the frequency of occurrence of the most frequently appearing words in these scientific articles. Notably, the terms that appear in the analysis correspond only to the root of the words to avoid semantic duplications and redundancies that may interfere with the final results.

The optimal number of clusters was established as 5 after the k values were analyzed on the silhouette graphics (Supplementary Figures S1 and S2). After the information of each cluster was reviewed, Cluster 2 was selected for further steps. This cluster contains topics related to skin, dermocosmetic applications, extracts and antioxidants (Figure 2 and Supplementary Figure S3). Although this cluster comprises the smallest number of articles, it represents a highly specific and thematically aligned subset, as illustrated in Supplementary Figures S5 and S6. The subsequent clusters exhibit a divergence in their thematic focus relative to Cluster 0, which primarily explored the impact of polyphenols on a variety of diseases. Cluster 1 delved into a more detailed botanical characterization of the extracts under consideration, while Cluster 3 delved into the realm of traditional medicine. Cluster 4, on the other hand, encompassed a less specific range of terms that did not align neatly with the classifications observed in the preceding clusters (Supplementary Figure S4).

**Figure 3:**
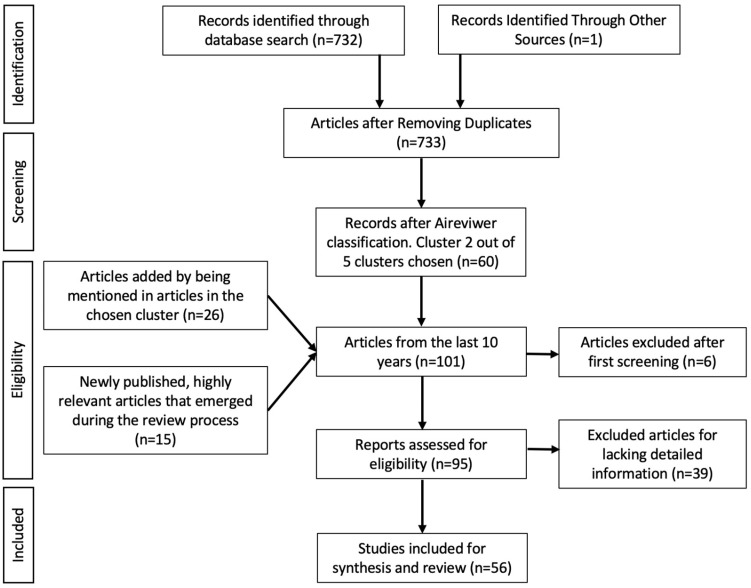
Diagram of the process of searching, screening and selecting articles for the present bibliographic review.

To ensure the most current and comprehensive analysis, our review process involved several steps. Initially, we retrieved articles from the designated cluster. To ensure a focus on contemporary research, our selection was limited to articles published within the past 10 years and classified by clusters and publication years, as illustrated in Supplementary Figure S7. Next, we implemented an initial exclusion process on the basis of the availability of full-length articles and their language, including only articles in English or Spanish. This step allowed us to narrow the pool of articles for further examination. Additionally, we incorporated articles that were cited within the clustered articles, as well as recent, highly relevant articles that fell outside the AI-driven selection but were deemed essential for inclusion. This approach was deemed necessary because of the diverse range of reviews and articles within the group, many of which extensively referenced other valuable works, as well as the continuous emergence of new publications during the review process. By incorporating these cited articles, we aimed to ensure that our analysis was thorough and inclusive. Ultimately, a total of 93 articles were carefully selected for analysis. Next, we further refined this selection by excluding articles that lacked sufficient evidence to support contrasting arguments. We specifically prioritized articles that provided detailed information on the specific activities being studied and the compounds responsible for these activities. This elimination process allowed us to focus on the 54 most relevant and informative articles for our review (Figure 3).

## 3. Main Characteristics of Pomegranate and Its Molecular Components

The pomegranate, scientifically referred to as *Punica granatum* L., is a shrub-like plant affiliated with the Lythraceae family. It is indigenous to North Africa and the Caucasus region, and it is also native to the Mediterranean region, both on the European and African shores. Moreover, it has been extensively dispersed to other areas, including the southern United States and various Asian countries. Owing to its widespread cultivation, the pomegranate is now readily accessible worldwide. This plant can reach a height of up to 10 m, featuring trunks coated in cracked bark that tends toward a grayish or brownish hue. While the leaves are initially red, they transition to a green color as they mature. The slender, lance-shaped leaves possess a glossy, leathery texture [20].

The pomegranate fruit is almost perfectly round and varies in size. Its exterior displays a crimson-red hue, encased by a yellowish inner skin with a leathery, layered texture. The vibrant red color of the fruit is primarily attributed to the presence of anthocyanins, which are abundant in the arils. These arils are the edible grains of the pomegranate, each consisting of a juicy, pigmented pulp that envelops a small, rigid seed, which together constitute the aril. Internally, the fruit is divided into compartments by thin, membranous walls, each filled with arils that may appear in red, pink, or white hues. It is commonly believed that the most flavorful pomegranates are those cultivated in regions with high temperatures [20]. Traditionally, pomegranates have been used for medicinal purposes, serving as remedies for persistent cough, intestinal worms, diarrhea, dysentery, and other health problems [21].

### Main Bioactive Components

Pomegranates contain a wide variety of phytochemicals with proven biological activity (Table 1). Pomegranate bioactive molecules have been shown to have strong antioxidant anti-inflammatory activities, and anticancer, antibacterial, and antiobesity effects have been described, mainly mediated through effective inhibition of oxidative stress and reductions in the activity of ROS, nitric oxide synthase (NOS), caspase-3, DNA fragmentation, and apoptosis, among other molecular mechanisms [7]. Among the most interesting active ingredients found in pomegranate, its polyphenols include molecules from the flavonoid, anthocyanidin and tannin subfamilies [1].

A total of 43 compounds were identified. Among these compounds, 51% were found in seeds, 21% in the peel, 18% in the fruit, and 5% in flowers. It should be noted that the term ‘fruit’ is used to categorize studies where the specific anatomical parts used for extraction were not explicitly detailed. The most referenced compounds were punicalagin and ellagic acid, which are known for their high concentrations in pomegranate. Interestingly, compounds identified in the peel were also found in the fruit, albeit at lower concentrations, making the peel a particularly intriguing area of study.

The quantitative prevalence of these compounds varies significantly across different tissues. In the juice and fruit fraction, organic acids dominate the nonvolatile profile, with citric acid acting as the primary constituent, representing up to 88% of the total organic acid content, followed by malic acid. However, the therapeutic and industrial value is largely concentrated in the polyphenolic richness of the peel and seeds. For instance, punicalagin, the most abundant ellagitannin, is present in large amounts in the peel, with concentrations ranging from 114.6 to 282.8 mg/g depending on the extraction method. This high concentration of hydrolysable tannins, combined with a significant fatty acid fraction in the seeds (comprising 25.8% of the identified diversity), underscores the fruit’s multifaceted chemical composition.

With respect to the overall chemical wealth of the fruit, ellagitannins and organic acids stand out as the most abundant groups. Specifically, punicalagin and ellagic acid represent the dominant polyphenolic fraction, whereas citric and malic acids constitute the primary organic acid content, establishing the foundational chemical profile of the fruit.

Among all the compounds identified and listed in Table 1, 43% were polyphenols. These include ellagitannins, gallotannins, anthocyanins, flavanol-anthocyanin adducts, flavonoids, phenolic acids, and other phenols. Particularly notable among these polyphenols are the ellagitannin subfamily, which includes hydrolysable tannins subsuming ellagic acid, punicalagin, punicalin, and pedunculagin [5,32].

## 4. Dermocosmetic Activities of Pomegranate Extracts

**Figure 4:**
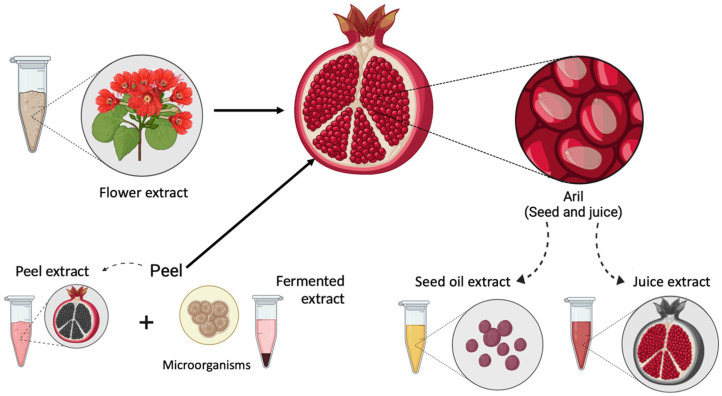
Schematic representation of the pomegranate and its natural sources of bioactive compounds. Different anatomical parts, including the flower, peel, aril (seed and juice), and seed oil, are highlighted as natural sources of various types of extracts.

Extracts can be obtained from different parts of the pomegranate, including the aril (seed and juice), peel, and flower, as shown in Figure 4. These extracts can exhibit various health-promoting properties [28] because of their bioactive components, as described in Table 1. While peel extracts are the most extensively studied because of their high density of ellagitannins, seed oil is characterized by its unique lipid profile, and juice or flower extracts offer specific combinations of anthocyanins and flavonoids. These diverse matrices function through distinct molecular mechanisms, ranging from oxidative stress modulation to the inhibition of extracellular matrix degradation enzymes. The distribution percentages presented in the following sections are derived from a systematic analysis of the literature specifically conducted for this review to quantify current research efforts. To provide a comprehensive overview, the following sections discuss these biological activities, highlighting the research trends and the prevalence of each extract type in the current scientific literature.

Studies reported in this work have shown that extracts from different parts of pomegranates have diverse potential dermocosmetic skin effects, such as inhibiting melanin synthesis, improving hydration and elasticity, preventing skin aging, and providing antioxidant and anti-inflammatory activity [35], among other effects. The dermocosmetic properties of different pomegranate extracts from different parts of the plant are summarized below.

### 4.1. Flower Extracts

Extracts derived from pomegranate flowers represent 9% of the current research. These studies often emphasize their specific polyphenolic composition, showing promising results in antiglycation activities and the prevention of skin aging.

Hydroalcoholic extracts obtained from pomegranate flowers have demonstrated antibacterial and wound-healing effects in an *in vivo* assay using rats, primarily mediated by ellagitannins [17]. A similar hydroalcoholic extract also exhibited inhibitory activity against elastase, collagenase, and tyrosinase enzymes [8], indicating potential for skin health applications. In addition to these direct extracts, another pomegranate flower extract, notably encapsulated in zein fibers by electrospinning and obtained through ultrasound-assisted extraction, showed significant antioxidant and antimicrobial effects in an *in vitro* assay [36]. These diverse effects were attributed mainly to a rich profile of compounds, including phenolic acids (such as gallic and ellagic acids), ursolic acid, triterpenoids (such as oleanolic, maslinic, and asiatic acids), flavonoids, and anthocyanins.

While extensive examination of pomegranate flower extracts in scientific literature has been relatively limited compared with that of the fruit, preliminary studies, as highlighted above, strongly indicate their distinct potential. Specifically, these studies have revealed promising wound-healing, antibacterial [17], and enzymatic inhibition properties unique to the flower’s composition or processing [8]. Although some of these characteristics, particularly antioxidant activity, are also commonly attributed to the fruit, the flower extracts offer a unique combination of compounds and specific effects, such as targeted enzymatic inhibition, that warrant further dedicated investigation for novel dermocosmetic applications [36].

### 4.2. Juice Extracts

Studies of pomegranate juice constitute 23% of the reviewed literature. Research in this area frequently highlights its rich content of anthocyanins and vitamin C, focusing on its synergistic effects on skin brightening and systemic antioxidant protection.

Conversely, research on pomegranate juice has focused predominantly on its rich polyphenol content, particularly ellagic acid, which is believed to contribute to numerous cosmeceutical applications. Studies have explored the potential benefits of pomegranate juice in treating skin conditions and protecting against UV radiation [13,37]. Most investigations have utilized oral supplementation as the primary method of delivery because of the known lack of toxicity associated with pomegranate fruit consumption.

The molecular mechanisms underlying the dermocosmetic effects of pomegranate juice include the modulation of oxidative stress pathways and melanogenic signaling [11]. Ellagic acid interferes with the nuclear factor erythroid 2–related factor 2 (Nrf2) signaling pathway, thus preserving redox homeostasis in skin cells [13]. Additionally, attenuation of mitogen-activated protein kinase (MAPK) and c-Jun signaling in UVB-exposed keratinocytes leads to decreased phosphorylation events, reducing photoaging [37]. Enzymatic inhibition of tyrosinase also plays a central role in the anti-pigmentation effects, supported by reduced melanocyte proliferation and melanin synthesis [38].

Pomegranate juice extract has been shown to have a broad range of skin health benefits through various formulations and mechanisms of action. One of the most consistently reported bioactive compounds across studies is ellagic acid, often found alongside punicalagins, flavonoids, and anthocyanins, which together contribute to the antioxidant, anti-inflammatory, and photoprotective properties of the extract.

Several studies have focused on its photoprotective and antipigmentation effects, mainly through the inhibition of melanogenesis. For instance, a hydroalcoholic extract of pomegranate juice, tested *in vitro* and *in vivo* in guinea pigs, showed a highly significant sun protection factor (SPF), photoprotective and depigmenting activity because ellagic acid can inhibit tyrosinase and melanocyte proliferation [11].

These findings align with results from a formulation of concentrated polyphenols evaluated *in vitro* in keratinocyte assays. The extract was shown to reduce UVB-induced phosphorylation of MAPK and c-jun proteins, lower intracellular glutathione (GSH) levels, and increase lipid peroxidation (LPO), contributing to strong antioxidant and photoprotective effects with a high SPF [37].

In addition to pigmentation, antiaging and anti-inflammatory properties have been widely documented. Freeze-dried pomegranate juice extract, tested *in vivo* in rats, significantly reduced skin inflammation by decreasing the infiltration of CD8+ T cells and macrophages, reducing the expression of proinflammatory cytokines such as TNF-α, and increasing antioxidant enzyme activity. It also modulated MHC class I and II expression, enhancing immune regulation and promoting wound healing and hydration [39]. A related study using supercritical fluid extraction (SFE) and *in vitro* assays confirmed the ability of the extract to modulate cytokine expression and preserve skin elasticity, attributing these outcomes to the presence of ellagic acid, punicalagins, quercetin, catechins, and anthocyanins [40].

Additionally, a formulation involving gold nanoparticles diluted in pomegranate juice and tested *in vitro* in fibroblasts and endothelial cells showed remarkable wound-healing and photoprotective effects. These effects were primarily mediated by ellagic acid, which modulated the Nrf2 pathway to preserve the redox balance of the skin [13], demonstrating that delivery systems can enhance or preserve the bioactivity of pomegranate components.

### 4.3. Seed Oil Extracts

Studies of seed-derived extracts and oils account for 20% of the literature. This section highlights the growing interest in its unique lipid profile, particularly that of punicic acid, which is primarily studied for its photoprotective, moisturizing, and anti-inflammatory properties.

Among the various components of pomegranate fruit, pomegranate seeds contain the greatest variety of compounds, including polyphenols and fatty acid compounds. While possessing similar properties to those of other pomegranate extracts, pomegranate seed extract has shown promising effects in reducing stretch marks [27], promoting hair growth [41], and suppressing fungal growth [7]. These findings collectively underscore the intriguing and auspicious nature of studying pomegranate seed extract.

The dermocosmetic effects of pomegranate seed oil can be attributed to both enzymatic inhibition and antioxidant pathways. Fatty acids in the extract inhibit key enzymes such as collagenase, elastase, and hyaluronidase, which are responsible for degrading collagen, elastin, and hyaluronic acid, respectively [7]. These actions help maintain the integrity of the extracellular matrix. Moreover, modulation of MMP-1 and MMP-3 expression via MAPK and JNK/p38 pathways further supports the preservation of skin structure. Antioxidative properties include the neutralization of reactive oxygen species (ROS), protection of cellular membranes, and prevention of UVB-induced DNA damage, all of which contribute to its high photoprotective efficacy [41].

Pomegranate seed oil has gained increasing attention because of its rich profile of bioactive compounds and its broad spectrum of biological effects, particularly in the context of skin health, inflammation, and oxidative stress. Various extraction methods have been employed to isolate and enhance its functional constituents, such as conjugated fatty acids (notably punicic acid), polyphenols, flavonoids, anthocyanins, tocopherols, and sterols, which contribute to its diverse pharmacological potential.

Soxhlet extraction of seed oils has revealed high concentrations of essential fatty acids, phenols, and sterols, which display potent antioxidant and anti-inflammatory activity *in vitro* [42]. These effects are in line with those observed in pomegranate juice, whose high phenolic and flavonoid contents, including gallic acid, ellagic acid, and anthocyanins, have been shown to reduce oxidative stress and modulate inflammatory markers in various cellular models.

Hydroalcoholic extracts of pomegranate seed oil, tested both *in vivo* (rats and humans) and *in vitro*, have shown strong anti-inflammatory, antifungal, and antibacterial activity. These effects are mediated by the inhibition of matrix-degrading enzymes such as collagenase, elastase, and hyaluronidase, which play a central role in skin aging and inflammation. Additionally, this extract modulates enzymes such as tyrosinase (linked to pigmentation) and reduces the expression of markers of oxidative stress (including MDA and nitric oxide). Notably, the inhibition of COX-2 also decreases the synthesis of proinflammatory prostaglandins, contributing to the protection of collagen integrity, skin elasticity, and overall dermal health [7].

Ultrasound-assisted extraction techniques, particularly liquid–liquid extraction, have been used to isolate polyunsaturated, monounsaturated, and saturated fatty acids from pomegranate seed oil, including high concentrations of punicic acid. *In vitro*, these extracts demonstrated antioxidant, estrogenic, and anti-inflammatory properties while enhancing the biosynthesis of collagen, elastin, and hyaluronic acid—crucial structural components of the extracellular matrix involved in maintaining skin firmness, elasticity, and hydration [43]. These findings further support the regenerative potential of pomegranate seed oil as a dermocosmetic active ingredient, closely aligning with similar observations from pomegranate juice polyphenols.

Nanoemulsion formulations of pomegranate seed oil have also shown enhanced biological activity because of improved bioavailability. *In vitro* studies using human erythrocyte assays demonstrated potent antioxidant and photoprotective effects, including inhibition of MMP-1 and MMP-3 via MAPK and JNK/p38 signaling pathways, as well as the neutralization of reactive oxygen species (ROS). These nanoemulsions significantly reduced UVB-induced DNA damage by decreasing thymine dimer formation, thereby increasing skin protection and suggesting a high natural SPF value [41]. Such photoprotective and antiaging properties have also been associated with pomegranate juice extracts, which offer similar benefits through their anthocyanin and ellagic acid contents.

Other *in vitro* investigations have focused on compounds isolated from pomegranate seed oil, such as oleamide, octadecenamides, squalene, and γ-tocopherol. These bioactive compounds downregulate the expression of key inflammatory and degradative mediators, including COX-2, lipid peroxidation, tyrosinase, hyaluronidase, elastase, collagenase, nitric oxide (NO), and nitric oxide synthase (NOS). The extensive modulation of enzymatic and oxidative pathways translates into anti-inflammatory, antioxidant, antimicrobial, and antiapoptotic effects [7], reinforcing the therapeutic potential of seed oil and being consistent with the key effects of pomegranate juice polyphenols in reducing the oxidative and inflammatory burden in various biological systems.

Furthermore, extracts obtained via ultrasound-assisted deep eutectic solvent (DES) extraction were particularly rich in anthocyanins, ellagic acid, and kaempferol. These compounds are also highly abundant in pomegranate juice. These extracts exhibited strong antioxidant, antimicrobial, and anti-inflammatory activities *in vitro* [44], highlighting once again the consistency of bioactivity across different pomegranate-derived matrices and extraction techniques.

Taken together, the evidence highlights the significant pharmacological overlap between pomegranate seed oil and pomegranate juice extract, particularly in terms of their contents of phenolic compounds, fatty acids, and antioxidant molecules. Regardless of the extraction method employed, hydroalcoholic, ultrasound-assisted, or nanoformulated, pomegranate seed oil is a potent source of bioactive agents with promising applications in skin repair, antiaging, photoprotection, and anti-inflammatory therapy.

### 4.4. Peel Extracts

Research on peel extracts represents the dominant trend in this field, accounting for 41% of the selected studies. This prevalence is primarily because of the high concentration of punicalagin and its versatile application in antioxidant, antiaging, and wound healing formulations, making it the most explored matrix in pomegranate dermocosmetic research.

The pomegranate peel extract is composed of compounds similar to those found in pomegranate juice, albeit in a more concentrated form because of its lower water content. Notably, the peel represents a valuable source of bioactive molecules that might otherwise be discarded as an agricultural byproduct. This makes it particularly attractive for translational research focused on sustainable and cost-effective raw materials. Studies have demonstrated that polyphenolic compounds such as ellagic acid can be efficiently extracted from high-purity peel material, highlighting its dermocosmetic potential [45]. To enhance its stability and bioavailability, encapsulation techniques or carrier systems are often employed in extract formulations.

Among the principal constituents of pomegranate peel, punicalagin is known for its diverse medicinal properties. Experimental evidence supports its antioxidant, antibacterial, antiallergic, anti-inflammatory and regeneration effects, as in a study by Hayouni et al. (2011) [46], where a topical ointment containing pomegranate peel extract was evaluated for its wound-healing efficacy in guinea pigs. Daily application over 10 days resulted in marked improvements in wound contraction, epithelial regeneration, and tissue remodeling. These outcomes were accompanied by notable antioxidant, antibacterial, and antifungal activities, suggesting that the extract not only promotes tissue repair but also supports a protective microenvironment conducive to healing [46]. Similarly, Yan et al. (2013) [47] investigated the effect of a polyphenol-rich pomegranate peel gel on incisional wounds in diabetic rats. Treated animals showed accelerated wound closure, increased fibroblast infiltration, enhanced collagen regeneration, and improved vascularization at the wound site, indicating that the extract actively supports key phases of the healing process even under impaired conditions [47]. To further support these findings, Fleck et al. (2016) [48] evaluated the effects of a magisterial formulation of ethanolic pomegranate peel extracts on chronic nonhealing ulcers in guinea pigs. The extract promoted visible improvements in tissue recovery, which aligns with prior evidence of its regenerative potential in compromised wound models [48].

From a mechanistic standpoint, pomegranate peel extracts function through multiple pathways. Enzymatic inhibition plays a prominent role, with downregulation of the activity of tyrosinase, elastase, and hyaluronidase contributing to reduced pigmentation and preservation of skin elasticity [29]. The peel also shows marked antioxidant activity through the scavenging of free radicals (DPPH, ABTS, and NO), inhibition of lipid peroxidation, and enhancement of superoxide dismutase (SOD) activity. Immunomodulatory effects include downregulation of inflammatory cytokines (TNF-α, IL-1β, IL-6, and IL-8) and a reduction in monocytic cell adhesion [21,30,46]. Furthermore, peel extracts promote fibroblast migration and proliferation, support wound healing, and increase the expression of skin barrier genes such as glutathione peroxidase (GPX) and SOD1, reinforcing hydration and barrier function [49].

Pomegranate peel extracts have a wide range of beneficial effects on skin health, primarily because of their high content of bioactive compounds such as punicalagin, ellagic acid, gallic acid, and various polyphenols and flavonoids. They include wound healing, antioxidant, anti-inflammatory, antipigmentation, and photoprotective effects.

Several studies highlight the efficacy of pomegranate peel for wound healing. Hydroalcoholic extract, notably rich in polyphenols, promoted fibroblast migration and proliferation in rats *in vivo*, leading to increased collagen production, skin regeneration, vascularization, and epithelialization [47]. Similarly, a hydrogel containing punicalagin from pomegranate peel exhibited *in vivo* wound-healing effects in chronic ulcers in both model rats and humans [48]. Furthermore, a methanol extract of pomegranate peel, containing punicalagin and ellagic acid, demonstrated antioxidant activity by inhibiting lipid peroxidation and increasing the potency of free radical scavenging, contributing to wound-healing effects in an *in vivo* rat study [46]. An aqueous encapsulated extract has also been shown to enhance *in vitro* wound healing [50].

A hydroalcoholic extract formulated as a nanostructured lipid carrier (NLC) showed significantly enhanced *in vitro* permeation, and its ellagic acid content was linked to the inhibition of tyrosinase (via copper chelation), as well as antibacterial, anti-inflammatory, anti-tyrosinase, and antiallergic effects, all of which contributed to wound healing [29].

The antioxidant and anti-inflammatory properties of pomegranate peel have been consistently observed across various studies. Punicalagin and ellagic acid from a dry extract, which were assessed in an *in vitro* THP-1 cell assay, attenuated inflammatory cytokine secretion and cell adhesion of monocytic cells, highlighting their anti-inflammatory potential [21]. Another dry extract, this time from *Brassica oleracea L*. inflorescence vesicles but containing galloylglucose, punicalagin, ellagic acid, and gallic acid, offered protective effects against mutations in mtDNA induced by UV-B radiation in an *in vitro* keratinocyte cell assay, indicating antioxidant and photoprotective benefits [31]. Microwave-assisted extraction yielded ellagitannins (punicalagins and punicalins) and flavonoids (quercetin and catechins) from pomegranate peel, all of which exhibited antioxidant effects *in vitro* [51].

In addition to promoting wound healing and anti-inflammatory activity, pomegranate peel also offers significant photoprotective and anti-pigmentation benefits. Spray-dried herbosomes (phospholipid-based complexes) and montmorillonite nanocarriers (layered silicates) containing punicalagin (alpha and beta), ellagic acid, gallic acid, and punicalin showed *in vitro* antipigmentation and photoprotective effects with a high SPF [52]. Enzymatic hydrolysis of pomegranate peel produced ellagic acid, polyphenols, and flavonoids, which reduced collagen breakdown by MMPs; increased the expression of HAS1 and FLG (improving skin barrier function); and increased the gene expression of GPX and SOD1 while decreasing IL-1β levels and inhibiting MAPK signaling. This multifaceted action conferred antioxidant, anti-inflammatory, and photoprotective effects (a high SPF) and reduced collagen degradation [49]. Ultrasound-assisted alkaline hydrolysis extracts containing ellagic acid, punicalagin, gallic acid, caffeic acid, and p-coumaric acid also demonstrated antioxidant effects *in vitro* [53].

Last, a liposomal hydroalcoholic extract, comprising polyphenols and anthocyanins, was found to reduce changes in hair color ex vivo in dyed hair assays [54]. Punicalagin and EGCG from pomegranate peel, in an *in vitro* keratinocyte cell assay, decreased the mRNA levels of TNF-α, IL-1β, IL-6, and IL-8 and attenuated particulate matter with a diameter of 10 μm or less (PM10)-induced ROS production and the expression of NOXs, inflammatory cytokines, and MMP-1, indicating that cell viability was rescued and that inflammatory responses were attenuated [30].

### 4.5. Fermented Extract

A specialized niche identified in recent research is the study of fermented pomegranate extracts, which accounts for 6% of the studies. This emerging trend focuses on how fermentation enhances the bioavailability of compounds such as urolithins, improving their efficacy in skin microbiome balance and enhancing antiaging effects.

Fermented pomegranate extracts have demonstrated a range of beneficial effects superior to traditional extracts. For instance, fermentation using *Lactobacillus plantarum* has been shown to increase the concentration of ellagic acid and total phenols, providing significant protection against oxidative stress by reducing reactive oxygen species (ROS) and inhibiting the expression of matrix metalloproteinases (MMPs) like MMP-1 in human skin fibroblasts [55].

Further research into fermented pomegranate extracts, specifically those produced via solid-state fermentation (SSF) with *Aspergillus niger* in an *in vitro* assay, revealed additional promising attributes. This method facilitated the enzymatic release of bound polyphenols and their structural transformation (e.g., hydroxylation and oxidation), including the conversion of punicalagin to ellagic acid by hydrolysis and lactonization. The active compounds identified in this extract included gallic acid, ellagic acid, isoquercetin, and protocatechuic acid. Consequently, this SSF-derived extract exhibited significant antioxidant, antiaging, anti-inflammatory, and photoprotective effects, including a high SPF [56].

These findings collectively underscore the potential of various fermentation methods to enhance the beneficial properties of pomegranate extracts, particularly in relation to their antioxidant capabilities, antiaging effects, and skin health benefits, through the enrichment and transformation of key bioactive compounds such as gallic acid, ellagic acid, and anthocyanins.

## 5. *In Vivo* Human Studies Supporting the Dermocosmetic Effects of Pomegranate

As an overview of the mentioned studies using different pomegranate extracts, this section focuses exclusively on clinical trials conducted in humans. These *in vivo* studies provide essential data regarding the practical dermocosmetic applications of pomegranate-derived compounds. One of the key therapeutic targets evaluated across several human studies is wound healing, a multifaceted biological process involving inflammation, tissue regeneration, and remodeling, which is often compromised in chronic wounds or during skin aging.

In this context, various clinical studies have investigated the therapeutic potential of pomegranate extracts, with particular emphasis on their bioactive compounds, mainly punicalagin, ellagic acid, urolithins, punicic acid, and polyphenols. These compounds exert effects through multiple mechanisms, including enzymatic inhibition of antioxidant activity, modulation of cellular signaling pathways and promotion of dermal regeneration.

Among the most robust findings, Chakkalakal et al. (2022) [32] conducted a randomized, double-blind, placebo-controlled trial using Pomella^®^, an oral pomegranate peel extract standardized to 30% punicalagin. This treatment significantly reduced wrinkle severity and modulated the skin microbiome, increasing the levels of *Staphylococcus epidermidis* and *Bacillus* spp. A decreasing trend in transepidermal water loss (TEWL) and sebum excretion was also observed, attributed to the antioxidant and anti-inflammatory properties of punicalagin, as well as its activation of the Nrf2 pathway and inhibition of tyrosinase, which are relevant to skin tone modulation [32].

Similarly, Henning et al. (2019) [5] reported that both PomX (a pomegranate extract rich in ellagic acid and punicalagin) and PomJ (pomegranate juice containing high levels of anthocyanins and ellagitannins) exhibited potent photoprotective effects in human subjects. These included an increase in minimal erythema dose (MED) and a high level of sun protection factor (SPF) against UVB-induced skin damage, along with modulation of the skin microbiome. These protective effects were primarily attributed to the ability of ellagic acid to attenuate MAPK/c-jun pathway activation and inhibit lipid peroxidation, thereby mitigating oxidative stress and inflammation. In support of these findings, an additional study using a dry pomegranate extract confirmed similar SPF-enhancing and microbiome-modulating effects. These effects were attributed to the synergistic activity of ellagic acid, punicalagin, and urolithins, which together inhibited melanin synthesis, suppressed matrix metalloproteinases (MMPs), and stimulated the production of extracellular matrix components such as collagen type I and hyaluronic acid [5].

Moreover, López-Ríos et al. (2021) [33] evaluated a botanical hydroalcoholic multiherbal formulation containing pomegranate peel extract enriched in polyphenols, particularly ellagic acid and punicalagin, in menopausal women. The observed improvements focused on menopause-specific health domains and were tested in response to oral supplementation; these improvements included antioxidant, anti-inflammatory, immunomodulatory, and even neurotransmission-modulatory properties through the enhancement of serotonergic activity, suggesting broader health-related quality-of-life improvements, especially for menopausal women [33].

Other studies have focused on nutraceutical formulations. For instance, Emanuele et al. (2017) [38] demonstrated that a blend including pomegranate extract standardized in ellagic acid improved skin hydration, elasticity, and barrier function. These outcomes align with the known effects of ellagic acid on fibroblast proliferation and collagen synthesis and the upregulation of the expression of antioxidant enzymes such as superoxide dismutase (SOD) and glutathione peroxidase (GPx), which are critical for dermal repair and redox balance [38].

Topically, pomegranate seed oil, rich in punicic acid and minor phenolic compounds, has demonstrated anti-inflammatory and wound-healing properties. Pilot studies have shown enhanced fibroblast migration and collagen regeneration, confirming its role in re-epithelialization and tissue remodeling [7]. Additionally, when combined with *Croton lechleri* resin, topical application reduced the appearance of striae distensae (stretch marks), likely due to the synergistic effects of conjugated fatty acids, flavonoids, and anthocyanidins on dermal remodeling [27].

Oral formulations combining pomegranate juice with other botanicals, such as *Leucotomos*, also yielded synergistic outcomes in humans. This supplementation resulted in reduced erythema, melanin, and sebum production. The underlying mechanisms included COX-2 downregulation, tyrosinase inhibition, hyaluronan synthesis, and inhibition of dermal-degrading enzymes such as elastase, collagenase, and MMP-1 [38].

A particularly innovative strategy to enhance pomegranate bioactivity involves fermentation [56]. This process enables enzymatic transformations, such as hydroxylation, oxidation, and lactonization, which convert punicalagin into the more bioavailable and potent ellagic acid. Fermented extracts not only exhibit increased antioxidant and anti-inflammatory activities but also stimulate hyaluronic acid production and water retention, which are crucial for hydration and elasticity, making them attractive for antiaging therapies [55]. Consistent with this systemic approach, the biological relevance of the pomegranate extends to its microbe-derived metabolites, specifically urolithins [32]. Urolithin A (UA) has emerged as a key player in skin homeostasis because it enhances mitochondrial function through the induction of mitophagy. This process ensures the clearance of damaged mitochondria, thereby reducing the production of mitochondrial reactive oxygen species (ROS) and maintaining cellular energy balance in dermal fibroblasts, which ultimately counteracts cellular senescence and promotes skin longevity [55].

In this context, Chan et al. (2022) [55] investigated a fermented pomegranate peel extract obtained through biotransformation with *Saccharomyces cerevisiae* and *Lactobacillus plantarum*. This study demonstrated promising effects on skin hydration, brightness, and elasticity, as well as antioxidant, antiaging, and depigmenting properties. These effects were attributed to the presence of fermentation-enhanced bioactive compounds, including anthocyanins, catechins, and gallic acid, which exhibited free radical scavenging activity (DPPH, ABTS+, NO), tyrosinase inhibition, and promotion of hyaluronic acid synthesis. While the evidence regarding its antiaging efficacy remains preliminary, the findings suggest a compelling avenue for future research focusing on fermentation-derived metabolites capable of modulating key molecular pathways involved in cellular aging, thereby reinforcing the multifunctional potential of fermented pomegranate extracts in dermocosmetic applications [55].

## 6. Discussion

Analysis of the reviewed literature and the compiled data highlights the broad spectrum of dermocosmetic benefits associated with pomegranate compounds and their derived products. These benefits include antioxidant, antibacterial, anti-inflammatory, wound healing, hydration, collagen-preserving, antiaging, photoprotective (high SPF), and protective effects against environmental stressors. The efficacy of these effects is largely attributed to the presence of diverse bioactive phytochemicals, particularly polyphenols such as ellagitannins, flavonoids and anthocyanins, along with essential fatty acids [14,43]. Unlike previous manual reviews, our systematic approach powered by AIReviewer allowed for the objective processing of a large volume of literature (732 articles), identifying emerging trends that emphasize the importance of microbe-derived metabolites in skin health. A primary example of this shift is the antiaging potential of UA, which is significantly bolstered by its role in countering cellular senescence. By restoring mitochondrial health and preventing the senescence-associated secretory phenotype (SASP) through mitophagy, UA promotes a youthful dermal environment by improving collagen synthesis and reducing the expression of matrix metalloproteinases (MMPs). This mechanistic insight, highlighted by our analysis, positions UA as a cornerstone metabolite in the current research landscape of pomegranate-derived bioactive compounds [16,55].

While recent comprehensive reviews, such as that by Dimitrijevic et al. (2024), have effectively summarized the effects of pomegranate on healthy and diseased skin through traditional narrative methods, our work provides a distinct methodological advancement [16]. By utilizing AIReviewer, we were able to systematically screen and objectively categorize a significantly larger volume of literature (732 articles). This approach minimizes selection bias and allows for the identification of emerging bioactive clusters and specific molecular trends, such as the precise role of urolithins in mitochondrial health, which may be less emphasized in manual reviews. Thus, our study complements the literature by providing a more rigorous, data-driven framework that defines the current state of the art in pomegranate-based dermocosmetics.

Among polyphenols, ellagitannins, especially punicalagin, play a critical role in preserving collagen structure and skin elasticity. They exert potent antioxidant effects, limiting oxidative damage and supporting dermal architecture. Ellagic acid, the active hydrolysis product of punicalagin, has been extensively studied for its wound-healing and hydration-enhancing properties. It accelerates tissue repair by stimulating fibroblast migration and proliferation and contributes to improved skin moisture retention through the upregulation of skin barrier genes such as HAS1, GPX, and SOD1. Additionally, flavonoids such as quercetin and catechins support anti-inflammatory and anti-aging activities by modulating cytokine expression, mitigating oxidative stress, and preserving the extracellular matrix [39,40,49].

Essential fatty acids found in pomegranate seed oil, particularly punicic acid, contribute to the maintenance of skin health by modulating inflammatory responses and supporting structural integrity. The dermal benefits are better contextualized as promoting skin resilience and reducing the impact of environmental insults such as UV radiation and pollution. These lipophilic compounds inhibit matrix-degrading enzymes such as collagenase, elastase, and hyaluronidase, thereby preserving skin firmness and elasticity [7,41].

Anthocyanins, which are highly abundant in pomegranate juice and peel, further increase the skin’s defense through photoprotective effects. They increase the natural SPF of the skin, mitigate UVB-induced oxidative stress, and inhibit MAPK/c-Jun signaling pathways, which are central to photoaging and inflammation [31,49]. These molecules also reduce erythema and pigmentation, making them valuable components in formulations targeting sun damage and uneven skin tone [32,38].

**Figure 5:**
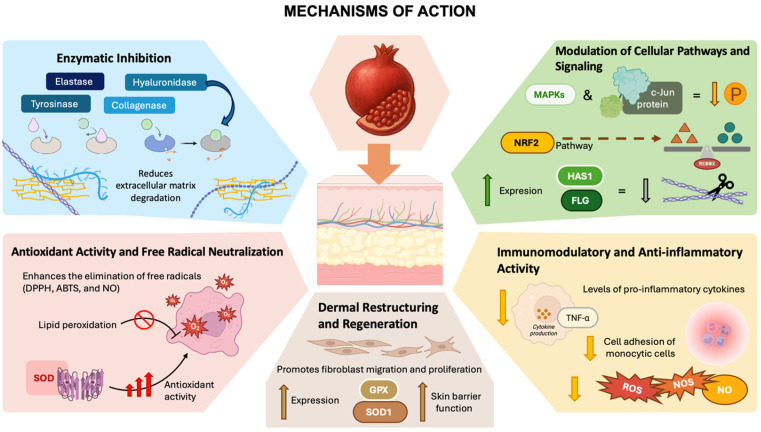
Main dermocosmetic mechanisms underlying the effects of pomegranate on human skin physiology.

At the molecular level, the dermocosmetic activity of pomegranate-derived compounds arises from the orchestration of several synergistic mechanisms (Figure 5). These include enzymatic inhibition (of collagenase, elastase, hyaluronidase, and tyrosinase), redox regulation through Nrf2 activation, and anti-inflammatory signaling via MAPK, JNK/p38, and downstream cytokine suppression (e.g., TNF-α, IL-6, IL-1β, and IL-8). Additionally, they support skin regeneration through fibroblast stimulation and matrix remodeling, which collectively enhance skin barrier repair and hydration [7,13,37,38,41].

Antioxidant mechanisms are particularly robust in pomegranate extracts. Pomegranate polyphenols scavenge ROS and RNS, inhibit lipid peroxidation, and upregulate endogenous antioxidant enzymes such as SOD and GPX [7,39,46]. This antioxidant reinforcement delays the onset of cellular senescence and supports a more youthful dermal phenotype. In parallel, anti-inflammatory pathways are modulated through reduced expression of adhesion molecules and cytokines, thereby minimizing redness, irritation, and inflammatory lesion formation [21].

Crucially, this youthful phenotype is further sustained by the activity of microbe-derived metabolites, specifically urolithins [16,32]. UA has emerged as a key player in skin longevity because it enhances mitochondrial function through the induction of mitophagy. This selective autophagy process ensures the clearance of damaged mitochondria, thereby reducing the production of mitochondrial reactive oxygen species (ROS) and maintaining cellular energy balance in dermal fibroblasts. By restoring mitochondrial quality control and preventing the senescence-associated secretory phenotype (SASP), UA directly counteracts cellular aging, positioning pomegranates as a comprehensive ‘longevity’ ingredient in advanced skincare [32,55].

The wound healing properties of peel and seed extracts, which promote dermal regeneration by enhancing fibroblast migration, collagen synthesis, and neovascularization, are particularly notable. These effects are especially pronounced in compromised skin models, where pomegranate extracts significantly improve tissue repair and structural recovery [7,47].

Recent studies have also shown that fermentation of pomegranate peel enhances its dermocosmetic potential. The bioconversion of punicalagin into ellagic acid via microbial fermentation leads to increased antioxidant capacity and bioavailability. This process also promotes the release of minor phenolics and the structural transformation of polyphenols via hydroxylation and lactonization. Topically applied fermented extracts have demonstrated the ability to improve hydration, brightness, and elasticity of the skin, offering a novel strategy to increase the efficacy of natural formulations [55,56].

**Figure 6:**
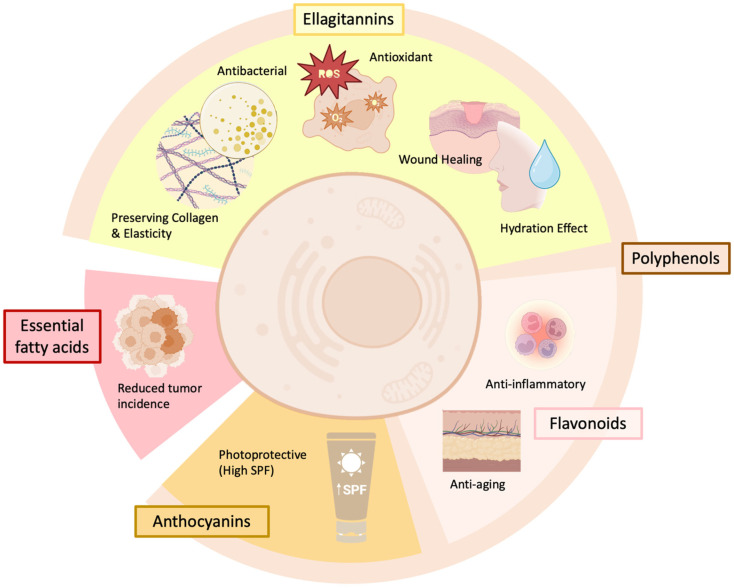
Main dermocosmetic properties observed in human cells for pomegranate extracts and the key families of natural compounds responsible.

These findings highlight the multifunctional potential of pomegranate-derived bioactive compounds in dermocosmetic formulations, offering a broad range of benefits for skin protection, rejuvenation, and repair. All of these effects are visually represented in Figure 6, which illustrates the key bioactive compounds in pomegranate extracts and their associated dermocosmetic properties.

In light of these multifunctional effects and given the favorable safety profile of pomegranate-derived compounds, future research should prioritize the development of advanced delivery systems to optimize their dermocosmetic potential. Encapsulation technologies such as nanoemulsions, liposomes, and polymeric nanoparticles offer promising strategies to increase the stability, bioavailability, and skin penetration of key phytochemicals [29,31,41,52,54]. These approaches could enable the creation of next-generation formulations with improved efficacy and targeted action, which is particularly beneficial for managing chronic skin conditions, photodamage, and age-related dermal deterioration.

Compared with other gold-standard botanical extracts commonly used in skincare, pomegranate offers a uniquely multifaceted profile. While green tea (*Camellia sinensis* (L.) Kuntze) is renowned for its EGCG-driven antioxidant capacity and *Centella asiatica* (L.) Urb for its wound-healing properties, pomegranate provides superior synergistic effects in terms of photoprotection and matrix preservation [37,38]. Unlike resveratrol or curcumin, which often face stability challenges in topical formulations, pomegranate ellagitannins and their metabolites demonstrate potent multitarget inhibition of collagenase and elastase. These findings position *P. granatum* as a more versatile alternative to traditional extracts such as licorice, rosemary, or melissa, especially in formulations targeting extrinsic aging and chronic inflammation [8,16].

## 7. Future Perspectives and Conclusions

As scientists delve deeper into how pomegranate extracts interact with the skin, as outlined in the articles referenced within this review, personalized skincare solutions may become a reality. Implementing sustainable extraction techniques will ensure the preservation of high product efficacy while reducing the ecological footprint through the repurposing of byproducts or waste materials, such as pomegranate skins or seeds, which have numerous compelling applications within the realm of dermocosmetics. The market for the pomegranate-infused dermocosmetic sector is fueled by consumer preferences for environmentally friendly and organic products, consequently fostering the advancement of a circular economic model.

The challenges encountered revolve around the susceptibility of pomegranate extracts to degradation when they are exposed to light, heat, and oxygen, consequently decreasing their stability and efficacy in formulations. Additionally, research is focused on exploring potential formulations, encapsulation techniques, or carriers that can enhance the properties of pomegranate extracts. The identification of economically feasible solutions is paramount for preserving the quality of products without inflating the cost of dermocosmetics.

In conclusion, pomegranate emerges as a highly versatile fruit, endowed with manifold advantages that extend beyond its mere consumption in various forms. Ongoing research has recently produced intriguing findings regarding the extract derived from pomegranate skin and the essential oils extracted from its seeds. The realm of pomegranate study still holds a vast territory waiting to be explored and subjected to investigation, particularly in terms of novel avenues for innovation. These include the potential utilization of nanosystems and the fermentation of pomegranate extracts, which not only improve bioavailability but also open the door to discovering new metabolites with superior bioactivity.

Furthermore, future research should investigate the potential of pomegranate-derived compounds in the management of immune-mediated skin diseases, such as psoriasis and atopic dermatitis. Another critical goal is the exploration of UA to regulate skin energy metabolism, improve barrier function, and reduce age-related dermal deterioration. Future research could particularly benefit from investigating specific nanosystems, such as nanoliposomes or nanoemulsions, to increase the stability of the labile compounds identified in Table 1 or to improve the transdermal penetration of metabolites such as UA, thereby maximizing their efficacy in topical applications for both dermocosmetic and therapeutic purposes.

## Figures and Tables

**Table 1:** Main bioactive phytochemicals identified in pomegranate, their molecular structure and chemical classification, some of the plant parts in which they are most abundant and biological effects.

Molecule	Structure	Type of Molecule	Plant Part	Reference
3,3′,4′-Tri-O-methylellagic acid	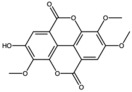	Ellagitanin	Fruit	[22]
5-Hydroxymethylfurfural	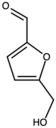	Furanic compound	Seed oil	[7]
7-Hexadecenal, (Z)-	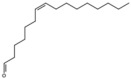	Unsaturated fatty acid	Seed oil	[7]
9-Octadecenoic acid, methyl ester, (E)-	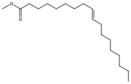	Unsaturated fatty acid	Seed oil	[7]
9,12-Octadecadienoic acid (Z,Z) (linoleic acid)	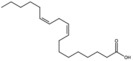	Polyunsaturated fatty acid	Seed oil	[7]
17-Alpha-estradiol (estradiol)	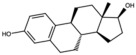	Steroidal estrogen	Seed oil	[1]
Caffeic acid	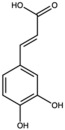	Phenolic acid	Fruit	[23]
Catechin	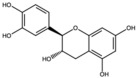	Flavonoid	Juice	[23]
Chlorogenic acid	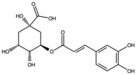	Phenolic acid	Peel	[23,24]
Cis-11-Eicosenamide	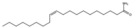	Unsaturated fatty acid	Seed oil	[7]
Conjugated linoleic acid (CLA)	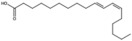	Conjugated fatty acid	Fruit	[1]
Coumaric acid	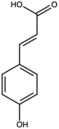	Phenolic acid	Peel	[25]
Coumestrol	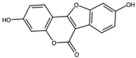	Phytoestrogen	Dry seed	[26]
Cyanidin	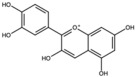	Anthocyanidin	Seed oil	[27]
Daidzein	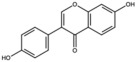	Isoflavone	Dry seed	[26]
Delphinidin	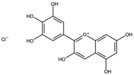	Anthocyanidin	Seed oil	[27]
Dodecyl acrylate	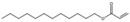	Acrylic ester	Seed oil	[7]
Ellagic acid	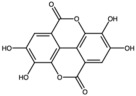	Ellagitannin	Fruit and peel	[1,28,29]
Epigallocatechin- 3-gallate (EGCG)	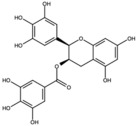	Catechin	Peel	[24,30]
Erucic acid	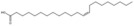	Monounsaturated fatty acid	Seed oil	[7]
Estrone	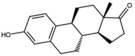	Steroidal estrogen	Seed oil	[1]
Ethyl brevifolincarboxylate	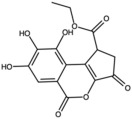	Ellagitannin	Fruit	[22]
Ferulic acid	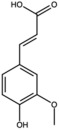	Phenolic acid	Peel	[25]
Gallic acid	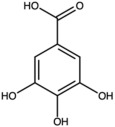	Phenolic acid	Peel	[25]
Galloylglucose	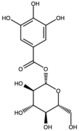	Gallotannin	Fruit	[31]
Genistein	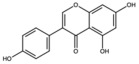	Isoflavone	Dry seed	[26]
Hexadecanamide (palmitic acid amide)	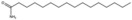	Saturated fatty acid	Seed oil	[7]
Kaemperol	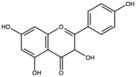	Flavonoid	Peel	[29]
Maslinic acids	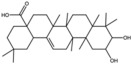	Terpene	Fruit	[22]
Myricetin	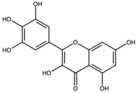	Flavonoid	Fruit	[23]
n-Hexadecanoic acid	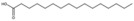	Saturated fatty acid	Seed oil	[7]
Octadecanoic acid	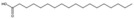	Saturated fatty acid	Seed oil	[7]
Octadecenamide	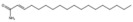	Saturated fatty acid	Seed oil	[7]
Oleamide	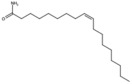	Unsaturated fatty acid	Seed oil	[7]
Oleanitrile	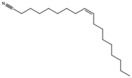	Unsaturated fatty acid	Seed oil	[7]
Oleanolic	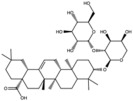	Triterpenoid	Flower	[32]
Pedunculagin	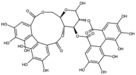	Ellagitannin	Fruit Peel (pericarp)	[32]
Pelargonidin	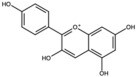	Anthocyanidin	Seed oil	[27]
Pentanoic acid, 5-hydroxy-, 2,4-di-t-butylphenyl esters	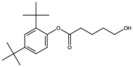	Phenolic ester of a fatty acid	Seed oil	[7]
Punicalagin	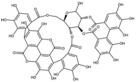	Ellagitannin	Fruit	[28,29,33]
Punicalin	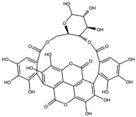	Ellagitannin	Peel	[29]
Quercetin	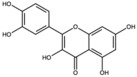	Flavonoid	Peel and juice	[25,29]
Rutin	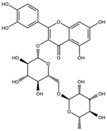	Flavonoid	Peel	[25]
Squalene	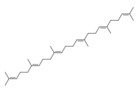	Hydrocarbon & terpene	Seed oil	[7]
Stigmas-3,5-diene	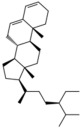	Terpene	Seed oil	[7]
Tocopherol	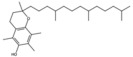	Polyphenol	Seed oil	[7]
Ursolic acid	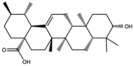	Triterpenoid	Flower	[32]
Vitamin C	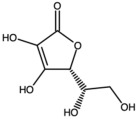	Water-soluble vitamin	Fruit	[1,34]

## Data Availability

The AIReviewer source code and detailed methodology can be accessed at: AIReviewer Pomegranate Cosmetic (https://drive.google.com/file/d/1pGn-yzJ4K-NqBnOeIUqQffwuOc4ImHZZ/view?usp=sharing, accessed on 2 April 2024).

## Supplementary Materials

The following supporting information can be downloaded at: https://www.mdpi.com/article/10.3390/antiox15030332/s1. Supplementary Figure S1. Silhouette analysis graph showing different scores for the number of clusters to assist in selecting the optimal clusterization. Supplementary Figure S2. Silhouette graphs for different numbers of clusters. Supplementary Figure S3. (A) Word cloud featuring the 75 most frequent words from the total articles analyzed. (B) Word cloud featuring the 75 most frequent words of Cluster 2. In both panels, a larger font size indicates a higher frequency of appearance; color variations are for visual differentiation only. Supplementary Figure S4. (A) Bar graph showing the number of articles present in each cluster. (B) Pie chart representing the percentage distribution of articles across clusters. Supplementary Figure S5. Frequency bar graphs showing the 15 most frequent words for: (A) Cluster 0, (B) Cluster 1, (C) Cluster 3, and (D) Cluster 4. Supplementary Figure S6. PCA plot of the articles across different clusters. Each point represents an article, with clusters differentiated by color. Supplementary Figure S7. (A) Line graph showing the distribution or evolution of articles by cluster. (Nota: Asegúrate de completar esta frase si el gráfico muestra algo específico como “over time” o “by year”).
