# Supplementary material for: Dermocosmetic Potential of Punica granatum: A Systematic Review of Bioactive Compounds and Skincare Applications
Source: Antioxidants (Basel). 2026 Mar 6;15(3):332. doi: 10.3390/antiox15030332 (PMC13024184; doi:10.3390/antiox15030332)
Supplement: Supplementary file 1 [file antioxidants-15-00332-s001.zip › antioxidants-4173034-supplementary.pdf]

Dermocosmetic Potential of *Punica granatum*: A Systematic Review of Bioactive Compounds and Skincare Applications

Nerea Pons-Rocamora [1], Enrique Barrajón-Catalán [1], María Herranz-López [1], Vicente Micol [1,2,*] and Francisco Javier Álvarez-Martínez [1]

Supplementary material

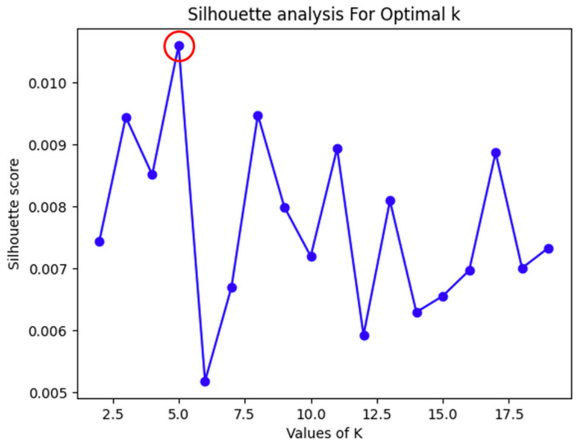

Supplementary Figure S1. Silhouette analysis graph showing different scores for the number of clusters to assist in selecting the optimal clusterization.

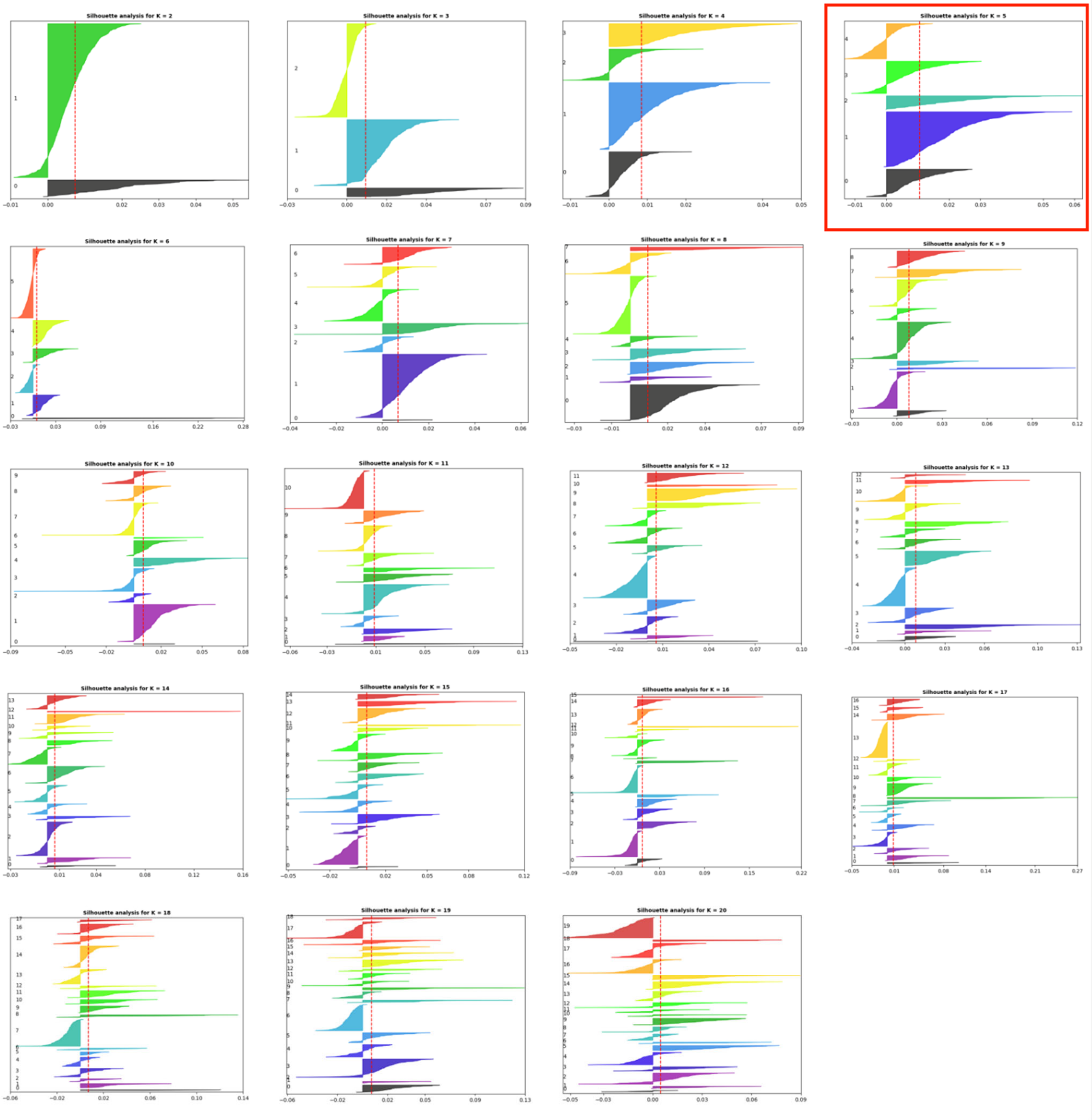

Supplementary Figure S2. Silhouette graphs for different number of clusters.

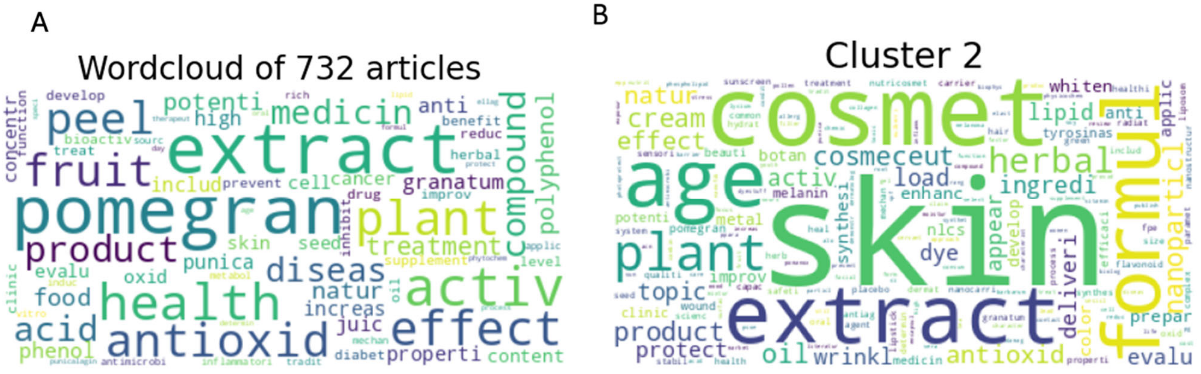

Supplementary Figure S3. (A) Word cloud featuring the 75 most frequent words from the total articles analyzed. (B) Word cloud featuring the 75 most frequent words of Cluster 2. In both panels, a larger font size indicates a higher frequency of appearance; color variations are for visual differentiation only.

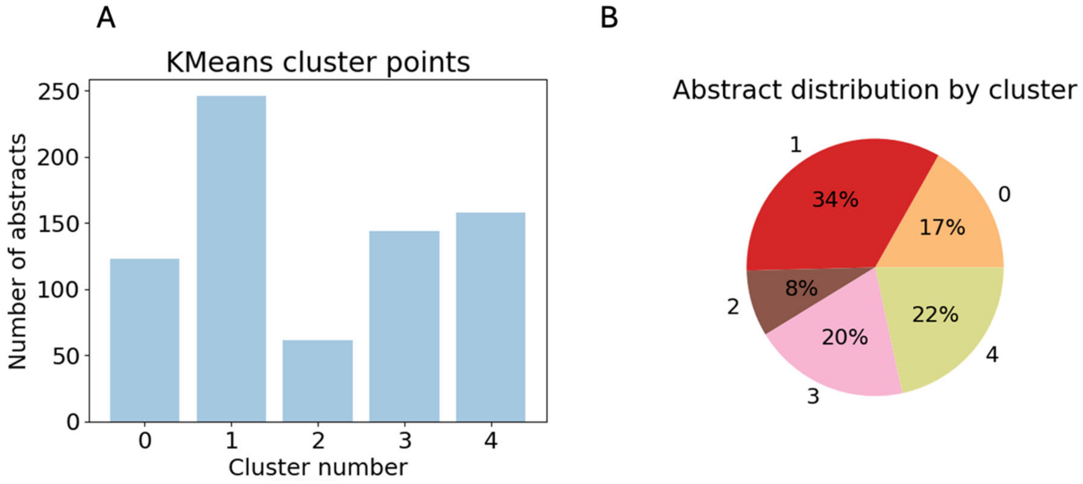

Supplementary Figure S4. (A) Bar graph showing the number of articles present in each cluster. (B) Pie chart representing the percentage distribution of articles across clusters.

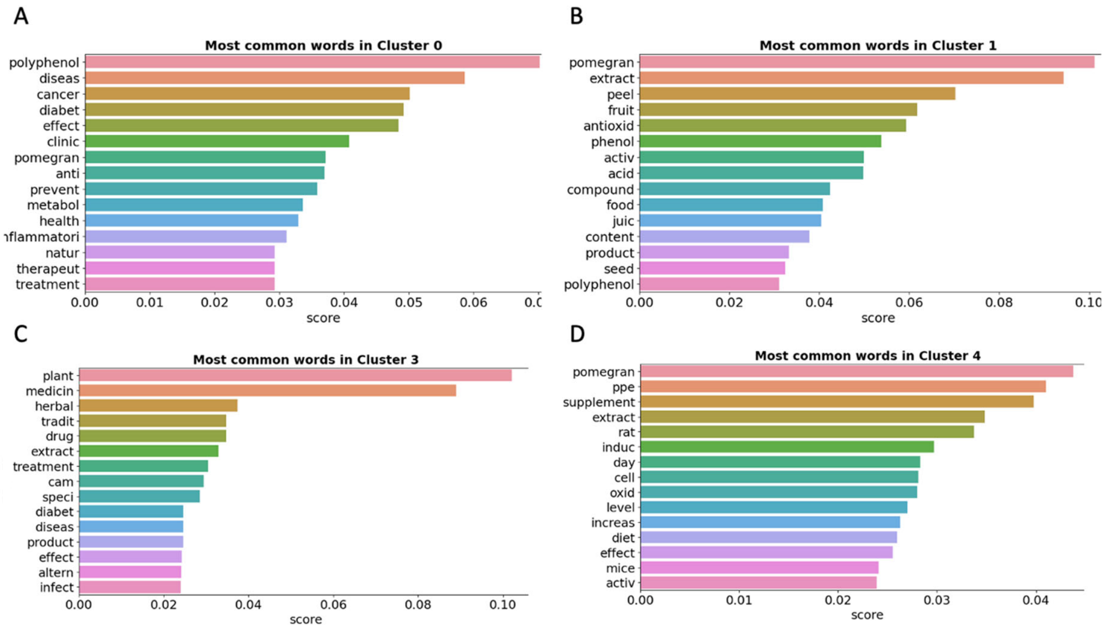

Supplementary Figure S5. Frequency bar graphs showing the 15 most frequent words for: (A) Cluster 0, (B) Cluster 1, (C) Cluster 3, and (D) Cluster 4.

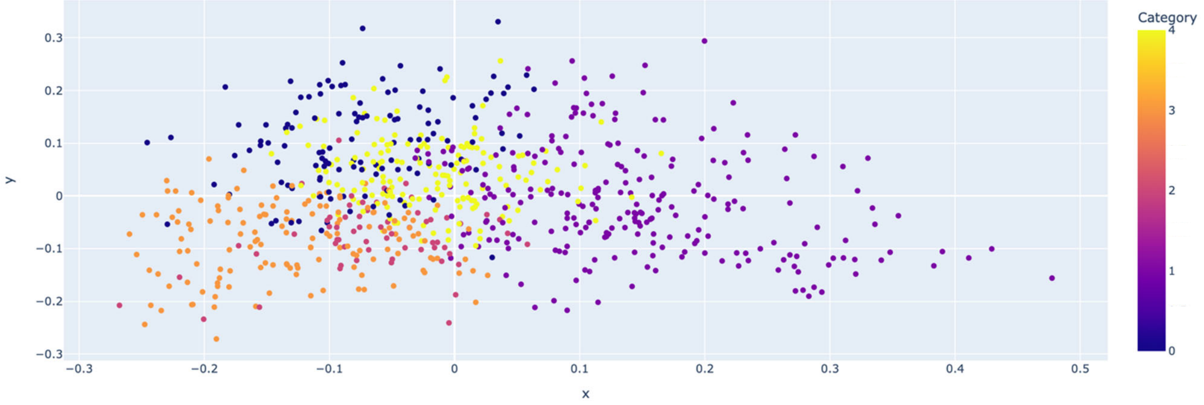

Supplementary Figure S6. PCA plot of the articles across different clusters. Each point represents an article, with clusters differentiated by color.

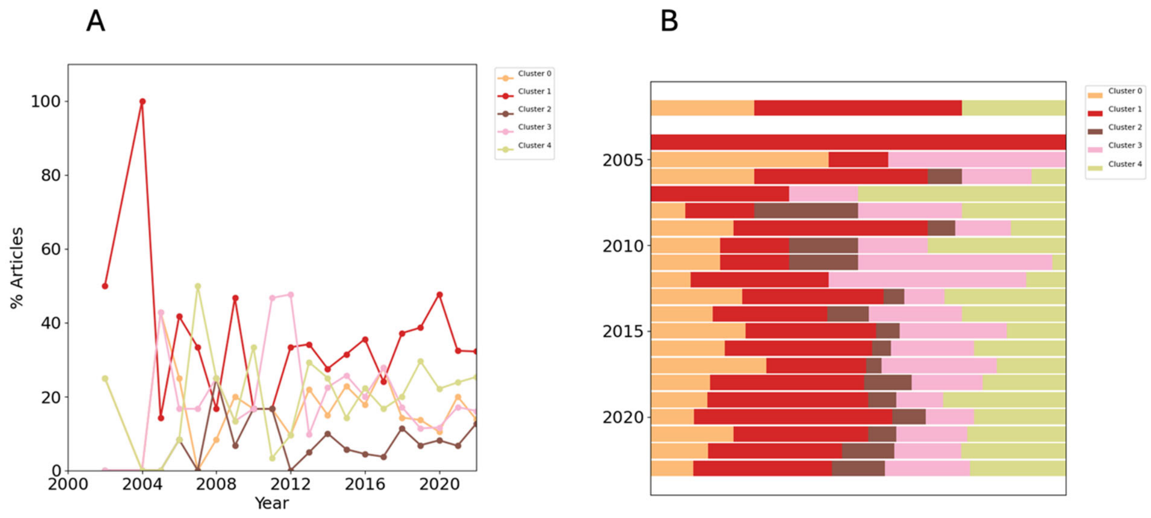

Supplementary Figure S7. (A) Line graph showing the distribution or evolution of articles by cluster. (*Nota: Asegúrate de completar esta frase si el gráfico muestra algo específico como "over time" o "by year")*.
